# Risk Factors for Pericardiocentesis After Paediatric Cardiac Surgery

**DOI:** 10.1016/j.cjcpc.2024.10.009

**Published:** 2024-11-06

**Authors:** Vikhashni Nagesh, Alyssa Chappell, Jesse Batara, Andrew S. Mackie

**Affiliations:** a Department of Pediatrics, University of Alberta, Edmonton, Alberta, Canada; b Women and Children’s Health Research Institute, University of Alberta, Edmonton, Alberta, Canada

## Abstract

**Background:**

Pericardial effusions are common after paediatric cardiac surgery and can lead to cardiac tamponade in a small minority. However, it is difficult to predict which patients with an effusion will require pericardiocentesis. Therefore, among children with a postoperative effusion, we sought to identify risk factors for requiring pericardiocentesis.

**Methods:**

We conducted a case-control study including paediatric patients who underwent cardiac surgery between January 1, 2005, and July 1, 2020, at the Stollery Children’s Hospital. Cases were defined as those who underwent pericardiocentesis within 2 months of cardiac surgery and were compared with controls who had an effusion but did not require pericardiocentesis. Controls were matched 2:1 to cases based on age and year of surgery.

**Results:**

There were 42 cases and 84 controls. The median age at surgery was 3.0 years (interquartile range [IQR]: 0.5-6.4 years) among cases and 2.2 years (IQR: 0.4-5.8 years) among controls. The median weight at surgery was 13.5 kg (IQR: 6.4-18.0 kg) among cases and 13.5 kg (IQR: 4.9-23.1 kg) among controls. The use of anticoagulation or antiplatelet agents (odds ratio [OR]: 3.6, 95% confidence interval [CI]: 1.5-8.2, *P* < 0.01) in the postoperative period was independently associated with effusions requiring drainage. The use of prednisone postoperatively (OR: 3.3, 95% CI: 0.8-14.0, *P* = 0.10) and a history of previous pericardial effusion (OR: 4.7, 95% CI: 0.9-25.6, *P* = 0.08) were associated with a higher odds of pericardiocentesis but did not reach statistical significance.

**Conclusions:**

The use of postoperative anticoagulation was independently associated with the need for pericardiocentesis. Type of surgical procedure was not associated with the need for drainage.

## Résumé

**Contexte:**

L’épanchement péricardique est fréquent après une intervention chirurgicale en pédiatrie et peut provoquer une tamponnade cardiaque chez une petite minorité de patients. Il est néanmoins difficile de prédire quels sont, parmi les patients qui présentent un épanchement, ceux qui nécessiteront une péricardiocentèse. Nous avons donc cherché à repérer les facteurs de risque justifiant une péricardiocentèse chez les enfants présentant un épanchement postopératoire.

**Méthodologie:**

Nous avons réalisé une étude cas-témoins chez des enfants ayant subi une chirurgie cardiaque entre le 1^er^ janvier 2005 et le 1^er^ juillet 2020 au Stollery Children's Hospital d’Edmonton. Les cas étaient des patients ayant subi une péricardiocentèse dans les deux mois suivant une intervention chirurgicale cardiaque. Ils ont été comparés à des témoins ayant présenté un épanchement sans nécessiter de péricardiocentèse. Les témoins ont été appariés aux cas selon un rapport de 2 pour 1 en fonction de l’âge et de l’année de la chirurgie.

**Résultats:**

L’étude comptait 42 cas et 84 témoins. L’âge médian au moment de l’intervention chirurgicale était de 3,0 ans (écart interquartile [EI] : 0,5 à 6,4 ans) parmi les cas et de 2,2 ans (EI : 0,4 à 5,8 ans) parmi les témoins. Le poids médian au moment de l’intervention chirurgicale était de 13,5 kg (EI : 6,4 à 18,0 kg) parmi les cas et de 13,5 kg (EI : 4,9 à 23,1 kg) parmi les témoins. Un lien indépendant a été établi entre l’utilisation d’anticoagulants ou d’antiplaquettaires (risque relatif approché [RRA] : 3,6; intervalle de confiance [IC] à 95 % : 1,5 à 8,2; *p* < 0,01) en période postopératoire et les épanchements nécessitant un drainage. L’utilisation de prednisone en période postopératoire (RRA : 3,3; IC à 95 % : 0,8 à 14,0; *p* = 0,10) et les antécédents d’épanchement péricardique (RRA : 4,7; IC à 95 % : 0,9 à 25,6; *p* = 0,08) ont été associés à une plus forte probabilité de péricardiocentèse sans que cette association atteigne la signification statistique.

**Conclusions:**

Un lien indépendant a été établi entre l’utilisation d’anticoagulants en période postopératoire et la nécessité de recourir à une péricardiocentèse. Aucun lien n’a été établi entre le type d’intervention chirurgicale et le besoin de drainage.

Pericardial effusions are a commonly recognized complication in the postoperative period after cardiac surgery.^1^ Pericardiocentesis is the standard of care for the treatment of large, haemodynamically significant pericardial effusions.^2^ Other procedures may also be used to evacuate pericardial effusions, including open surgical drainage. Cardiac tamponade and invasive procedures are associated with morbidity and therefore best avoided when possible. Recent studies have documented the prevalence of postoperative pericardial effusions ranging from 10% to 53%.^3^, ^4^, ^5^ A small subset of these effusions are large and require pericardiocentesis or surgical drainage.^1^^,^^6^

There have been several paediatric studies describing risk factors for pericardial effusions and also for hospital readmission for the management of postoperative effusions.^3^, ^4^, ^5^, ^6^, ^7^, ^8^, ^9^ However, identification of risk factors for the largest effusions requiring pericardiocentesis or other invasive procedures has yet to be studied in children or adults. Early identification of high-risk patients may help clinicians optimize management in the postoperative period. Therefore, the objective of this study was to identify risk factors that predispose paediatric cardiac patients with an effusion to require pericardial drainage in the postoperative period.

## Methods

### Study design

We conducted a single-centre matched case-control study. Retrospective chart review data were collected from inpatient and outpatient medical records. Study data were collected and managed using REDCap electronic data capture tools hosted and supported by the Women and Children’s Health Research Institute at the University of Alberta.^10^

### Study population

The Western Canadian Children’s Heart Network Interprovincial Referral database was used to identify and stratify patients to the different arms of this retrospective study. The Western Canadian Children’s Heart Network is a specialized network dedicated exclusively to the treatment of children with heart conditions and connects 5 paediatric hospitals across 4 Canadian provinces.^11^ Cases and controls were children younger than 18 years who underwent a cardiac surgical procedure between January 1, 2005, and July 1, 2020, at the Stollery Children’s Hospital in Edmonton, Canada. Of note, the method of screening for effusions and management of effusions at our centre has not changed significantly during the study period.

Cases were defined as those who underwent pericardiocentesis, surgical pericardial drainage, or creation of a pericardial window within 2 months of cardiac surgery. We did not include cases who underwent emergent drainage within 24 hours of surgery as these were felt to be more likely to be related to surgical bleeding rather than an inflammatory process. Controls were defined as those who developed a pericardial effusion but did not require an invasive procedure to drain the effusion. Controls were matched 2:1 to cases based on age (with maximum age difference of 12 months) and year of surgery (±1 year).

### Predictor variables

Potential predictors that may predispose patients to require invasive interventions in the postoperative period were chosen based on a review of paediatric and adult literature.^3^^,^^5^^,^^6^^,^^8^^,^^9^^,^^12^, ^13^, ^14^, ^15^, ^16^, ^17^, ^18^ Predictor variables were sex;^3^^,^^12^^,^^13^ type of congenital heart disease;^3^^,^^5^^,^^8^^,^^9^^,^^14^ type of surgical intervention; presence of a positive postoperative nasopharyngeal aspirate;^6^ medications used in the postoperative period potentially to treat the pericardial effusion including steroids^15^ (oral or intravenous), diuretics,^12^ and nonsteroidal anti-inflammatories;^16^ and use of anticoagulation,^3^^,^^5^^,^^14^^,^^17^^,^^18^ excluding heparin for maintaining central venous catheter patency. Effusion size was obtained from echocardiographic reports and classified as trivial, small, moderate, or large. Among cases or controls having an effusion that became larger over time, the largest size of the effusion at any time point within 2 months of surgery was documented.

### Statistical analysis

Continuous variables were reported as medians with interquartile range. Categorical variables were reported as proportions. Univariate conditional logistic regression was used to analyse variables associated with the need for pericardial drainage. Four variables with *P* < 0.25 were explored further in a multivariable conditional logistic regression model. Given that there were 42 cases and needing a 10:1 ratio of cases to controls,^19^ we limited the multivariate analysis to 4 variables. Statistical analysis was performed using SAS Version 9.4 (SAS Institute Incorporated, Cary, NC).

## Results

The sample size consisted of 42 cases and 84 controls. Table 1 summarizes characteristics of cases and controls. Table 2 summarizes the size of effusions among cases and controls. Cases had larger effusions than controls; 100% of cases had echocardiograms reporting moderate-large effusions, relative to 15% of the controls.

**Table 1:** Descriptive characteristics

Characteristic	Cases (N = 42)	Controls (N = 84)
Sex		
Male	18 (43)	48 (57)
Female	24 (57)	36 (43)
Age at surgery (y)		
Median (IQR)	3.0 (0.5-6.4)	2.2 (0.4-5.8)
Weight at surgery (kg)		
Median (IQR)	13.5 (6.4-18.0)	13.5 (4.9-23.1)
Primary cardiac diagnosis category		
Single ventricle	16 (38)	14 (17)
Septal defects (ASD, VSD, and AVSD)	10 (24)	25 (30)
Other	16 (38)	45 (54)
Primary cardiac surgery category		
Septal defect closure	10 (24)	24 (29)
Norwood, Glenn + Fontan	12 (29)	14 (17)
Other	20 (48)	46 (55)
Previous cardiac surgery	9 (21)	18 (21)
Previous effusion	7 (17)	2 (2)
CPB use	35 (83)	76 (92)
NPA completed	12 (29)	14 (17)
Positive postoperative NPA	3 (7)	5 (6)
Postoperative medications		
Prednisone	10 (24)	3 (4)
Diuretics	38 (91)	75 (90)
NSAIDs	34 (81)	70 (83)
Anticoagulation	29 (69)	25 (30)
Unfractionated heparin	22 (52)	7 (8)
LMWH	18 (43)	11 (13)
Warfarin	8 (19)	8 (10)
ASA	14 (33)	13 (15)

Data are presented as N (%) unless otherwise specified.

ASA, acetylsalicylic acid; ASD, atrial septal defect; AVSD, atrioventricular septal defect; CPB, cardiopulmonary bypass; IQR, interquartile range; LMWH, low-molecular-weight heparin; NPA, nasopharyngeal aspirate; NSAID, nonsteroidal anti-inflammatory drug; VSD, ventricular septal defect.

**Table 2:** Pericardial effusion size as documented in echocardiogram reports

Pericardial effusion size	Cases (N = 42), N (%)	Controls (N = 84), N (%)
Trivial	0 (0)	14 (17)
Small	0 (0)	57 (68)
Moderate	11 (26)	13 (15)
Large	31 (74)	0 (0)

The univariate analysis is shown in Table 3. Patients receiving anticoagulants or antiplatelet agents had a significantly higher odds of undergoing pericardial drainage than those not receiving anticoagulants (odds ratio [OR]: 4.1, 95% confidence interval [CI]: 1.9-9.0, *P* < 0.01). Among the controls, 25 (30%) received 1 or more anticoagulants; 11 (13%) received low-molecular-weight heparin, 7 (8%) received unfractionated heparin, 8 (10%) received warfarin, and 13 (15%) received acetylsalicylic acid. Among the cases, 29 (69%) received anticoagulation, 18 (43%) received low-molecular-weight heparin, 22 (52%) received unfractionated heparin, 8 (19%) received warfarin, and 14 (33%) received acetylsalicylic acid. Patients receiving unfractionated heparin had a significantly higher odds (OR: 8.2, 95% CI: 3.1-21.8, *P* < 0.01) of undergoing pericardial drainage than those not receiving this type of anticoagulant. Likewise, patients receiving 2 or more anticoagulants had a significantly higher odds of undergoing interventions than those not receiving anticoagulants (OR: 6.2, 95% CI: 2.5-15.5, *P* < 0.01). There were 11 controls (13%) and 22 cases (52%) receiving more than 1 type of anticoagulation.

**Table 3:** Univariate analysis

Risk factors∗	Odds ratio	Odds ratio 95% CI		*P* value
		Lower limit	Upper Limit	
Sex, male vs female	0.5	0.2	1.2	0.11
Age at surgery (y)	1.1	0.9	1.5	0.34
Year of surgery	1.0	0.99	1.0	0.51
Steroids	4.6	1.4	14.7	0.01
Prednisone	6.7	1.8	24.2	<0.01
Other	1.3	0.2	8.0	0.75
Number of steroids: 1 vs 0	4.3	1.3	14.0	0.02
Diuretics	0.8	0.2	3.6	0.80
Furosemide	1.1	0.4	3.6	0.85
Spironolactone-hydrochlorothiazide	1.4	0.5	3.4	0.53
Spironolactone	2.7	0.8	9.5	0.11
Other	0.5	0.1	4.5	0.54
Number of diuretics: 1 vs 0	0.8	0.2	3.7	0.82
Number of diuretics 2+ vs 0	1.7	0.4	7.6	0.48
NSAIDs	0.7	0.3	2.1	0.59
Ibuprofen	0.6	0.2	1.5	0.26
Ketorolac	1.3	0.6	2.8	0.57
Number of NSAIDs: 1 vs 0	0.8	0.3	2.3	0.68
Number of NSAIDs: 2+ vs 0	0.9	0.3	2.9	0.85
Anticoagulants	4.1	1.9	9.0	<0.01
Unfractionated heparin	8.2	3.1	21.8	<0.01
Warfarin	2.3	0.7	7.5	0.16
ASA	2.5	1.0	6.3	0.05
Clopidogrel	No reported cases			
LMWH	4.5	1.8	11.7	<0.01
Other	No reported cases			
Number of anticoagulants: 1 vs 0	2.1	0.7	6.2	0.19
Number of anticoagulants: 2+ vs 0	6.2	2.5	15.5	<0.01
Cardiac diagnosis category vs single ventricle				
Septal defects (ASD, VSD, and AVSD)†	0.3	0.1	0.9	0.28
Other	0.3	0.1	0.7	0.07
Cardiac surgery category vs septal defect closure				
Norwood, Glenn + Fontan	2.2	0.7	7.0	0.11
Other	1.0	0.4	2.6	0.35
Previous cardiac surgery	1.0	0.4	2.5	1.00
Previous effusion	7.0	1.5	33.7	0.02
CPB use	0.4	0.1	1.4	0.16
Weight at surgery	1.0	0.97	1.1	0.62
Ventilation duration <1 d	0.5	0.2	1.3	0.16
Ventilation duration (d)	1.0	0.8	1.2	0.99
Drainage output				
POD1	0.9	0.9	1.0	0.15
POD2	0.9	0.9	1.0	0.16
POD3	0.9	0.7	1.0	0.12

ASA, acetylsalicylic acid; CI, confidence interval; CPB, cardiopulmonary bypass; LMWH, low-molecular-weight heparin; NSAID: nonsteroidal anti-inflammatory drug; POD, postoperative day.

∗ All yes vs no unless otherwise stated.

† Septal defect closure includes atrial septal defect (ASD), atrioventricular septal defect (AVSD), and ventricular septal defect (VSD).

Other variables associated with an increased odds of pericardiocentesis on univariate analysis included the postoperative use of prednisone (OR: 6.7, 95% CI: 1.8-24.2, *P* < 0.01) and a history of previous pericardial effusion (OR: 7.0, 95% CI: 1.5-33.7, *P* = 0.02) (Table 3).

On multivariable analysis (Table 4), the use of postoperative anticoagulants was associated with a higher odds of needing pericardiocentesis (OR: 3.6, 95% CI: 1.5-8.2, *P* < 0.01). The use of postoperative prednisone and a history of previous effusion were associated with an increased odds of pericardiocentesis but did not reach statistical significance.

**Table 4:** Multivariate analysis

Risk factors	Univariate		Model 1 multivariate		Final model multivariate	
	OR (95% CI)	*P* value	OR (95% CI)	*P* value	OR (95% CI)	*P* value
Prednisone	6.7 (1.8-24.2)	<0.01	4.5 (0.9-22.0)	0.07	3.3 (0.8-14.0)	0.10
Anticoagulants	4.1 (1.9-9.0)	<0.01	6.3 (2.0-20.4)	<0.01	3.6 (1.5-8.2)	<0.01
Previous effusion	7.0 (1.5-33.7)	0.02	4.5 (0.7-27.5)	0.10	4.7 (0.9-25.6)	0.08
Cardiac surgery category: Norwood, Glenn + Fontan vs septal defect closure	2.2 (0.7-7.0)	0.11	0.2 (0.04-1.4)	0.22	–	–

In the final multivariate model, the cardiac surgery category (Norwood, Glenn, and Fontan vs septal defect closure) was excluded from the analysis as it was not statistically significant in model 1.

CI, confidence interval; OR, odds ratio.

## Discussion

To our knowledge, this is the first study to assess risk factors for pericardial drainage among children having a postoperative pericardial effusion. The key findings of this study were that the use of anticoagulants postoperatively was associated with a higher odds of needing pericardial drainage.

Several risk factors for the development of postoperative pericardial effusion have been described in the literature. For example, certain surgical procedures confer an increased risk, including atrial septal defect repairs, repair of tetralogy of Fallot, heart transplantation, systemic-pulmonary shunts, Fontan surgery, and atrioventricular septal defect repairs.^3^^,^^5^^,^^6^^,^^8^^,^^9^ However, in this study, we did not identify a significant association between primary cardiac diagnosis or type of surgical procedure and the need for postoperative pericardiocentesis. Likewise, a history of previous cardiac surgery was not a risk factor for pericardiocentesis. The data regarding prior cardiac surgery are conflicting, with some authors reporting that this is associated with an increased^9^ risk of postoperative pericardial effusion and others reporting a decreased^7^ risk. However, ours is the first study to report on the risk of requiring drainage.

The medical treatment of pericardial effusions varies between centres and among clinicians and generally includes the use of nonsteroidal anti-inflammatories and corticosteroids.^20^ Corticosteroids as a treatment for pericardial effusions have been studied in the literature with some controversial findings. Some studies have shown that steroids are effective in the treatment of postoperative pericardial effusions in children.^15^^,^^21^ In contrast, other studies have shown no benefit.^7^^,^^22^ Despite the lack of high-quality paediatric literature, steroids are commonly used in the postoperative period for the management of pericardial effusions, particularly for patients having large effusions, as the pathophysiology of developing an effusion includes an inflammatory process in many patients.^23^ On univariate analysis, prednisone was associated with effusions requiring invasive interventions. We suspect that there was confounding by indication, as patients with the largest effusions are more likely to be treated with prednisone than those with smaller effusions. The retrospective nature of our study made it difficult to ascertain the indication for the use of prednisone in each case (ie, whether steroids were used to treat an effusion or for other reasons).

Anticoagulant therapy is commonly used in the postoperative period after paediatric cardiac surgery and is often used in valvular repairs/replacements and shunts (eg, Fontan and Blalock-Taussig shunts) as these operations confer increased thrombotic risk.^24^ Anticoagulation has been identified as a risk factor for the development of pericardial effusions and, in some cases, cardiac tamponade in the adult population.^14^^,^^17^^,^^18^ It is felt that in the anticoagulated state, bleeding into the pericardial space contributes to the accumulation of pericardial fluid; however, most effusions are serous or serosanguinous, rather than frank blood.^25^ Alternatively, blood in the pericardial space may induce an inflammatory response.^7^ We did not include cases who underwent emergent drainage shortly after surgery (within 24 hours of surgery) as these are often related to surgical bleeding.

There have been paediatric studies that identify warfarin specifically as a risk factor for developing pericardial effusion.^3^^,^^9^ To our knowledge, however, there have been no studies that identify unfractionated heparin or low-molecular-weight heparin as a risk factor. The use of multiple anticoagulants may lead to an excessively anticoagulated state, resulting in blood in the pericardial space. Our findings are consistent with those of Malouf et al.,^18^ who have reported that large postoperative effusions and tamponade are more likely to occur in anticoagulated patients,^26^ particularly those with evidence of excessive anticoagulation.^18^

### Limitations

This study has several limitations. Clinical decision-making around treatment of a pericardial effusion was variable among physicians and was not protocol based. The study was conducted at a single tertiary paediatric hospital and may not reflect clinical practice or outcomes in other programmes. There was a difference in the size of effusions between cases and controls (Table 2). Cases were documented to have effusion sizes that were primarily moderate or large, compared with controls that had no documented large effusions, which is relevant as large effusions are those most likely to need drainage.^1^^,^^2^ We did not consider effusion size to be a risk factor for pericardiocentesis, but rather a surrogate of the outcome.

## Conclusions

We identified that the use of anticoagulants in the postoperative period is associated with requiring drainage among patients with a pericardial effusion. The use of prednisone postoperatively and a history of previous effusion were associated with a higher odds of needing pericardiocentesis but did not reach statistical significance. The relationship between prednisone and pericardiocentesis is likely due to confounding by indication, rather than cause and effect. Children with an effusion who are on anticoagulants may benefit from more frequent echocardiographic monitoring in the postoperative period. Being judicious with the use of anticoagulation in the postoperative period may be beneficial in preventing the development of significant pericardial effusions requiring drainage. Further studies on the role of anticoagulants in the development of pericardial effusions are needed.

## References

[1] Weitzman L.B., Tinker W.P., Kronzon I. (1984). The incidence and natural history of pericardial effusion after cardiac surgery—an echocardiographic study. Circulation.

[2] Adler Y., Charron P., Imazio M. (2015). 2015 ESC Guidelines for the diagnosis and management of pericardial diseases: the Task Force for the Diagnosis and Management of Pericardial Diseases of the European Society of Cardiology (ESC) Endorsed by: the European Association for Cardio-Thoracic Surgery (EACTS). Eur Heart J.

[3] Cheung E.W.Y., Ho S.A., Tang K.K.Y. (2003). Pericardial effusion after open heart surgery for congenital heart disease. Heart.

[4] Clapp S.K., Garson A., Gutgesell H.P., Cooley D.A., McNamara D.G. (1980). Postoperative pericardial effusion and its relation to postpericardiotomy syndrome. Pediatrics.

[5] Dalili M., Zamani H., Aarabi-Moghaddam M. (2012). Pericardial effusion after pediatric cardiac surgeries: a single center observation. Res Cardiovasc Med.

[6] Galante G.J., Schantz D.I., Myers K.A. (2021). Echocardiographic screening for postoperative pericardial effusion in children. Pediatr Cardiol.

[7] Adrichem R., Le Cessie S., Hazekamp M.G. (2019). Risk of clinically relevant pericardial effusion after pediatric cardiac surgery. Pediatr Cardiol.

[8] Elias M.D., Glatz A.C., O’Connor M.J. (2017). Prevalence and risk factors for pericardial effusions requiring readmission after pediatric cardiac surgery. Pediatr Cardiol.

[9] Giordano R., Comentale G., Tommaso L.D. (2021). Pericardial effusion after pediatric cardiac surgery: a single-center study. Heart Lung.

[10] Harris P.A., Taylor R., Thielke R. (2009). Research Electronic Data Capture (REDCap)—a metadata-driven methodology and workflow process for providing translational research informatics support. J Biomed Inform.

[11] Western Canadian Children’s Heart Network Our organization/about WCCHN. https://wcchn.ca/.

[12] Imazio M., Brucato A., Rovere M.E. (2011). Contemporary features, risk factors, and prognosis of the post-pericardiotomy syndrome. Am J Cardiol.

[13] Lehto J., Kiviniemi T.O., Gunn J. (2016). Occurrence of postpericardiotomy syndrome admissions: a population-based registry study. Ann Med.

[14] Leiva E.H., Carreno M., Bucheli F.R. (2018). Factors associated with delayed cardiac tamponade after cardiac surgery. Ann Card Anaesth.

[15] Mizumoto M., Masaki N., Sai S. (2022). Efficacy of short-term oral prednisolone treatment in the management of pericardial effusion following pediatric cardiac surgery. Pediatr Cardiol.

[16] Imazio M., Spodick D.H., Brucato A., Trinchero R., Adler Y. (2010). Controversial issues in the management of pericardial diseases. Circulation.

[17] Tsang T.S., Barnes M.E., Hayes S.N. (1999). Clinical and echocardiographic characteristics of significant pericardial effusions following cardiothoracic surgery and outcomes of echo-guided pericardiocentesis for management: Mayo Clinic experience, 1979-1998. Chest.

[18] Malouf J.F., Alam S., Gharzeddine W., Stefadouros M.A. (1993). The role of anticoagulation in the development of pericardial effusion and late tamponade after cardiac surgery. Eur Heart J.

[19] Wilson VanVoorhis C., Morgan B. (2007). Understanding power and rules of thumb for determining sample sizes. Tutor Quant Methods Psychol.

[20] Somani N., Breur H. (2022). The efficacy of corticosteroids, NSAIDs, and colchicine in the treatment of pediatric postoperative pericardial effusion. Pediatr Cardiol.

[21] Wilson N.J., Webber S.A., Patterson M.W. (1994). Double-blind placebo-controlled trial of corticosteroids in children with postpericardiotomy syndrome. Pediatr Cardiol.

[22] Pasquali S.K., Hall M., Li J.S. (2010). Corticosteroids and outcome in children undergoing congenital heart surgery: analysis of the Pediatric Health Information Systems database. Circulation.

[23] Maranta F., Cianfanelli L., Grippo R. (2022). Post-pericardiotomy syndrome: insights into neglected postoperative issues. Eur J Cardiothorac Surg.

[24] Thom K.E., Hanslik A., Male C. (2011). Anticoagulation in children undergoing cardiac surgery. Semin Thromb Hemost.

[25] Ofori-Krakye S.K., Tyberg T.I., Geha A.S. (1981). Late cardiac tamponade after open heart surgery: incidence, role of anticoagulants in its pathogenesis and its relationship to the postpericardiotomy syndrome. Circulation.

[26] Pepi M., Muratori M., Barbier P. (1994). Pericardial effusion after cardiac surgery: incidence, site, size, and haemodynamic consequences. Br Heart J.

[27] Government of Canada Tri-Council Policy Statement: ethical conduct for research involving humans TCPS 2; 2022. https://ethics.gc.ca/eng/policy-politique_tcps2-eptc2_2022.html.

