# Supplementary material for: Risk Factors for Pericardiocentesis After Paediatric Cardiac Surgery
Source: CJC Pediatr Congenit Heart Dis. 2024 Nov 6;4(1):49–54. doi: 10.1016/j.cjcpc.2024.10.009 (PMC11955724; doi:10.1016/j.cjcpc.2024.10.009)
Supplement: Supplementary Material [file mmc1.pdf]

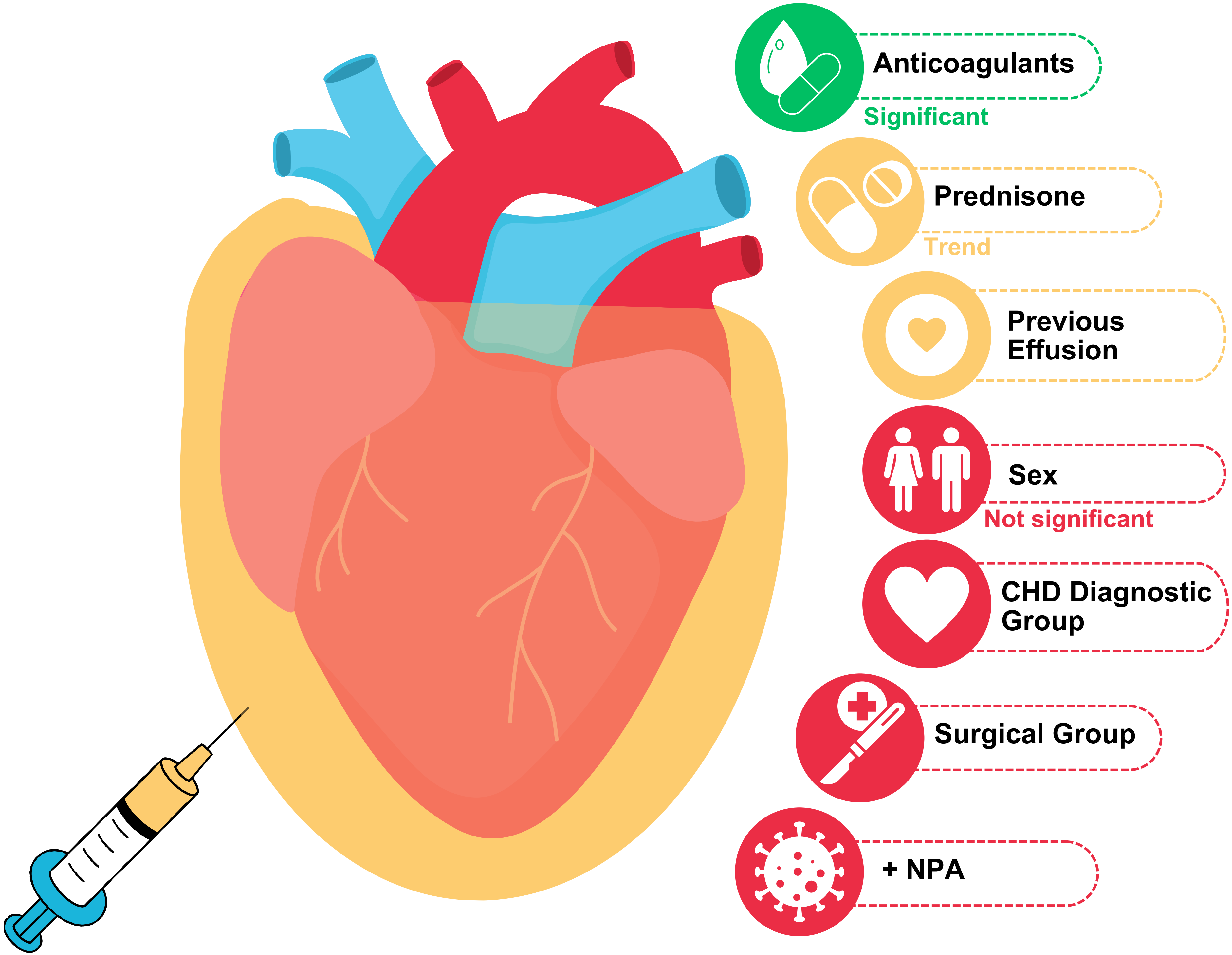
Anticoagulants
Significant
Prednisone
Trend
Previous Effusion
Sex
Not significant
CHD Diagnostic Group
Surgical Group
+ NPA
